# A Case of Successful Extirpation of the Parotid Gland

**Published:** 1877-08

**Authors:** J. M. Pace

**Affiliations:** Camden, Ark.


A Case of Successful Extirpation of the Parotid Gland.—*By J. M. Pace, M.D., Camden, Ark.*—In regard to the extirpation of this gland, a great many surgeons of eminence have doubted the practicability of the operation, and questioned the fact of its having ever been performed. Velpeau says, "should we take literally true, what authors of the last century have said of it, nothing could be more simple than the total eradication of the parotid gland. In our own times, on the contrary, nothing seems to be more uncommon—so much so, that numerous distinguished surgeons deny even its possibility." He also states that most of the cases reported are not conclusive, believing, with Boerhave and others, that in most instances, it was merely ablation of lymphatic tumors, developed in the depths of the parotid space. Allen Burns thought the operation impracticable. Cooper, however, says, to deny that the gland was ever extirpated, would be to impeach the veracity of some of our most eminent anatomists and surgeons; that no one will doubt the danger attending the operation; but to assert that it cannot be performed, is not only absurd, but not in keeping with the progress of the age.

Siebold professes to have extirpated the gland in 1796; Beclard in 1823; Gensoul, 1824; Prieger, 1826. Subsequently a host of other surgeons claim to have performed the operation. The late Dr. Geo. McClellan, of Philadelphia, is foremost in the ranks of American surgeons, having operated upon *eleven* cases with but one death.

Tumors—malignant and non-malignant—are often developed in the parotid gland, the nature of which it is often no easy matter to diagnose by merely superficial examination. Encephaloid tumors usually grow rapidly, attaining a large size. Scirrhus is more slow in its development, smaller in size, and very much more painful, though the latter symptom is not always pathognomonic of cancer; for even fibrous tumors often cause intense pain from pressure upon the surrounding nerves. Scirrhus tumors are usually solidly fixed, with ill-defined outlines; whereas, fibrous tumors are more movable and the mass is well defined.

The case that I have to report, as regards its malignant or non-malignant character, was hard to distinguish, not only because of the usual difficulty of diagnosing these morbid growths, but its extirpation had been attempted by another physician some months previously, which left a cicatrix and the surrounding integument in a glazed, purplish condition, and a tumor with ill-defined outlines, more or less resembling a malignant growth.

The subject was Miss L., aged about twenty-seven years, of a lymphatic temperament, general health good. Before proceeding to operate, I gave her in detail, all the results that might follow such an operation, and especially the facial paralysis, quoting from Prof. Gross, who states in his Surgery, that removal of the parotid is *always* followed by paralysis of the corresponding side of the face, in consequence of the division of the motor branch of the seventh pair of nerves. She was not to be frightened or deterred from the operation, but at once decided to have it out.

Accordingly, on the morning of the 26th of November last, she was placed upon the operating table, on her sound side, with her head and shoulders elevated. With the assistance of Drs. Bragg and Hudson, she was thoroughly anæsthetized by chloroform, when I proceeded to operate as follows: A single inscision was made in front of the ear extending from near the zygomatic arch to about one inch below the angle of the jaw; after this, the flaps were dissected up the entire length of the incision and the tumor loosened. I accomplished the rest of the operation by pulling and tearing with my fingers, aided with the handle of the knife, using the blade as little as possible. I, in fact, enucleated almost the entire gland by piece meals, beginning at the lower portion of the tumor and separating it upwards, for the reason that, should there be any hemorrhage, I could more easily control it, for there would be but one division of blood-vessels requiring ligation. Fortunately, there was but one small collateral branch of the temporo-maxillary artery divided, which was at once caught up and twisted, and it gave no further trouble. During the operation, I avoided the division of the facial nerve. After the extirpation of the gland, the digastric and stylohyoid muscles, and the external carotid were exposed, leaving but little doubt of the extirpation of the parotid gland. Fearing no further trouble from hemorrhage, the wound was closed with five interrupted sutures, and a few strips of adhesive plaster, after which cold water dressings were applied.

The second day after the operation, at her earnest solicitation, the patient was permitted to leave her bed; on the third day the sutures were removed. The wound healed by first intention.—*Virginia Medical Monthly.*

